# Mental Health and Social Support Are Key Predictors of Resilience in German Women with Endometriosis during the COVID-19 Pandemic

**DOI:** 10.3390/jcm11133684

**Published:** 2022-06-26

**Authors:** Roxana Schwab, Kathrin Stewen, Tanja Kottmann, Katharina Anic, Mona W. Schmidt, Tania Elger, Susanne Theis, Stefanie R. Kalb, Walburgis Brenner, Annette Hasenburg

**Affiliations:** 1 Department of Obstetrics and Gynecology, University Medical Center, Johannes Gutenberg University Mainz, Langenbeckstr. 1, 55131 Mainz, Germany; kathrin.stewen@unimedizin-mainz.de (K.S.); katharina.anic@unimedizin-mainz.de (K.A.); mona.schmidt@unimedizin-mainz.de (M.W.S.); tania.elger@unimedizin-mainz.de (T.E.); susanne.theis2@unimedizin-mainz.de (S.T.); stefanie.kalb@unimedizin-mainz.de (S.R.K.); walburgis.brenner@unimedizin-mainz.de (W.B.); annette.hasenburg@unimedizin-mainz.de (A.H.); 2 CRO Dr. med. Kottmann GmbH & Co. KG, 59077 Hamm, Germany; tk@cro-kottmann.de

**Keywords:** endometriosis, chronic pelvic pain, resilience, mental health, COVID-19 pandemic

## Abstract

Background: Endometriosis is a multifaceted chronic pain disorder that can have an impact on both physical and mental health. Women suffering from chronic pain may be more susceptible to various health disorders, especially during adversity, such as the COVID-19 pandemic. Previous research has identified resilience as a mediator between internal or external stressors and well-being. Methods: An online survey was conducted during the first wave of the COVID-19 pandemic in Germany through patient support groups of women with endometriosis. The Brief Resilience Score (BRS) was employed to evaluate resilience, while the PHQ-4 questionnaire was used to assess self-reported mental health. Univariate and multivariate logistic regression analyses were applied to determine resilience’s independent risk and protective parameters. Results: High educational level was found to be an independent supportive moderator of high resilience in women with a resilience score greater than the study population’s median (BRS > 2.66; OR 2.715; 95% CI 1.472–5.007; *p* = 0.001) but not in women in the highest resilience score quartile (BRS > 3.33). A decrease in perceived social support was detected to be the most powerful independent risk factor for low resilience: OR 0.541, 95% CI 0.307–0.952, *p* = 0.033 for predicting BRS > 2.66, and OR 0.397, 95% CI 0.189–0.832, *p* = 0.014 for predicting scores > 3.33 on the BRS scale. A high burden of mental health symptoms, as measured by the PHQ-4 scale, was negatively associated with resilience. Conclusions: Satisfying social support and good mental health were shown to be key resources for resilience. The results of this study may assist in the identification of women at risk for low resilience and the development of resilience-building strategies in patients with endometriosis.

## 1. Introduction

Endometriosis is a common chronic gynecological inflammatory disease caused by the growth of endometrial-like tissue outside the uterine cavity [1]. The induced chronic, inflammatory reaction of the abdominal cavity leads to a variety of unspecific symptoms, including dysmenorrhea, dyspareunia, non-cyclic pelvic pain, or infertility [1,2,3,4,5]. Additional symptoms, such as the deterioration of social and mental well-being [1,2,3,4,6], may arise in particularly vulnerable people as a result of a substantial diagnostic delay of up to 10.4 years in Germany [4,7]. Chronic pain has been identified as a risk factor for adverse mental outcomes [8], and permanent pelvic pain has been recognized as a predictor for poor psychological health and increased perceived stress in women with and without endometriosis [9,10,11,12,13,14]. Despite the presence of pain and pain-induced disability, some individuals with chronic pain appear to function normally.

Resilience is described as a trait, an outcome, or a process, depending on the definition [15,16]. It allows people to recover or bounce back from adversity or stressful events in their lives [15,17,18,19,20,21]. Numerous health-related outcomes, including less negative affect, more positive affect, fewer physical symptoms, and lower perceived stress, have been reported to be more present in highly resilient people [18,19,22]. In addition, resilience affects the perceived physical disability of patients with chronic pain, as well as the pain-related long-term morbidity and mortality [19,21,23]. Resilience was described as a predictor of threat coping and mental well-being in people who experienced natural disasters or health crisis [24,25,26,27,28].

Poor mental health, specifically self-reported depression or anxiety, and resilience mutually influence one other in women with chronic pain conditions [29,30,31]. The physiological and psychological response of the brain, the central organ in charge of the stress response to a stressor, often follows in an orchestrated manner. Inappropriate stress regulation may cause an increment in psychopathological risk [32,33,34]. Stress and resilience share common neurobiological response pathways [32,33,34] as well as mutual genetic and developmental influential factors [20]. The neurocircuitry is controlled and regulated for the maintenance of health and well-being under basal and stressful conditions in resilient individuals, who seem to adapt better to adverse environments and embrace stress as a challenge rather than a threat [32,33,34].

By the end of 2019, the novel severe acute respiratory syndrome coronavirus 2 (SARS-CoV-2) was first reported in China and rapidly spread to other countries by 2020. On 30 January 2020, the World Health Organization (WHO) announced the outbreak of a “public health emergency of international concern”, and on 11 March 2020, declared it to be a pandemic [35]. Various countries implemented public health measures, such as social distancing, quarantine, or economic lockdown, to prevent the spread of the virus [36], thus increasing the risk of social disconnection and loneliness. As a result, there has been a significant rise in mental health concerns, such as anxiety and depression, on the one hand, and lower resilience on the other, in populations worldwide [24,27,37,38,39]. Women in need of medical support, such as during pregnancy or the postpartum period, seem to be especially susceptible to a deterioration of physical and mental health [40,41,42,43]. During the COVID-19 pandemic, women with endometriosis reported a perceived decline in social support [7]. Chronic pain patients appear to be particularly vulnerable to the negative psychological impacts of social isolation, possibly because they entered the pandemic with fewer social resources than healthy controls [44].

Resilience is a dynamic trait and can change over time [16]. Resilience in women with endometriosis has received little attention to date [45]. Therefore, it is crucial to gain a better understanding of the mechanisms that contribute to the resilience of women with endometriosis to promote and to strengthen their ability to overcome the sometimes-deleterious pain-induced physical, social, and mental constraints. To date, resilience has been studied with a focus on particular life challenges or trauma, such as pain or pain-induced disability.

The purpose of this study was to examine the level of resilience in patients with endometrioses using the ultra-brief BRS questionnaire, which can be easily applied in daily life. Several predictors of resilience, including demographic characteristics [24], economic status [24,25,46], social factors or pain [16,47], and mental health [20], have already been identified; however, there may be other specific predictors in particular populations that are yet to be discovered.

To the best of our knowledge, this is the first study to assess the impact of sociodemographic aspects, disease-specific variables, pandemic-specific factors, and mental health on self-reported resilience in women with endometrioses during the COVID-19 pandemic. This approach enables us to examine concomitant factors that may contribute to resilience in women with endometriosis during difficult situations. Furthermore, our study aims to empower health care providers and family members to react more quickly and precisely in order to prevent negative health outcomes for patients with endometrioses in adverse situations.

## 2. Materials and Methods

### 2.1. Participants of the Study and Recruitment

An online questionnaire was distributed in endometriosis patient support groups between 6 April and 27 April 2020. In our survey, we followed the CHERRIES (Checklist for Reporting Results of Internet E-Surveys) checklist for comprehensive reporting quality [48]. A direct link to the survey and an invitation to participate were distributed through the Facebook platforms of patients’ support groups for endometriosis as an “open survey” within the mentioned target group. The data were collected anonymously using SoSciSurvey (a platform that stores all data in Germany and is subject to strict EU data protection laws), and no personal information, such as the IP address, was collected. Participation in the survey was voluntary, and no incentives were offered. Inclusion criteria were age older than 18 years, diagnosis of endometriosis during a surgical procedure, and informed consent to participate. Excluded from participating were only those who did not fulfill all the above-mentioned criteria. No other exclusion criteria, such as previous psychiatric comorbidities, were applied.

The questionnaire included questions related to demographic (age, being in a partnership, living alone, and educational level), disease (time since endometriosis diagnosis, age at diagnosis, endometriosis diagnostic delay, pain characteristics, pain intensity, and pain-induced disability), and pandemic (duration of social network reduction, being in isolation or quarantine, reduction in social contacts, and social support reduction during pain experience) parameters. An incremental five-point Likert scale was used to assess perceived social support by partners, family, and/or friends. The Likert scale variables “strongly disagree” and “disagree” on the one hand and “no change”, “agree”, and “strongly agree” on the other were clustered to evaluate the risk resulting from reduced social support compared with no change or increased social support.

Pain intensity was examined by the visual pain scale (VAS), a continuous scale ranging from 0 to 100 (100 being the strongest imaginable pain) [49]. Participants were asked to complete a questionnaire with their current pain intensity (VAS_C_) and their pain intensity and pain disability in the four weeks prior to the implementation of social distancing measures (VAS_P_), both being evaluated as a continuous variable.

Pain-induced disability was explored by the pain disability index (PDI). The PDI assesses the degree of pain-related disability in seven areas of daily life (family/home responsibilities, recreation, social activity, occupation, sexual behavior, self-care, and life-support activity), with each item being rated from 0 (no interference) to 10 (total interference). Sub-scores were created as basic activities (the sum of self-care and life-support activities) and discretional activities (all other activities). The global pain-induced disability (global PDI, total score comprising all items) can range between 0 and 70 [50]. Participants were asked to complete the questionnaire regarding their current pain-induced disability (PDI_C_) and their pain disability in the four weeks preceding the implementation of social distancing measures (PDI_P_). Pain-induced disability was evaluated as a continuous variable (PDI_P_, PDI_C_).

### 2.2. Brief Resilience Scale (BRS)

The BRS questionnaire was applied to examine the resilience of the study group. Smith et al. [17] developed the BRS to identify one’s ability to bounce back from stress. The BRS consists of six items graded on a five-point Likert scale: 1, 2, 3, 4, and 5 mean strongly disagree, disagree, neutral, agree, and strongly agree, respectively. Items 1, 3, and 5 are positively worded, while items 2, 4, and 6 are negatively worded. The average of all six items is used to calculate the score [51].

The psychometric properties of the German version of the BRS were investigated by Chmitorz et al. in two populations of healthy adults from the Gutenberg Brain Study and a second group representative of the adult German population [51].

### 2.3. Patient Health Questionnaire for Depression and Anxiety (PHQ-4)

The Patient Health Questionnaire for Depression and Anxiety (PHQ-4) was employed to determine the psychological burden of the study group. PHQ-4 is a combination of two items from the Patient Health Questionnaire for Depression (PHQ-2) and two items from the Generalized Anxiety Disorder Scale (GAD-2) [43]. PHQ-2 and GAD-2 scores ≥3 are cut-off points (“yellow flag”) between the normal range and probable cases of major depression or generalized anxiety, respectively, whereas PHQ-2 and GAD-2 scores of ≥5 are considered as “red flag” [52,53]. Löwe et al. recommended a PHQ-4 score of ≥6 as a “yellow flag” and a PHQ-4 score of ≥9 as a “red flag” for the presence of a depressive or an anxiety disorder [52,53].

### 2.4. Statistical Analysis

The study population was described using descriptive statistics (frequencies, means, and standard deviations) based on their responses to the BRS questionnaire. Differences between study respondents and non-respondents were examined by χ^2^-tests, the Fisher exact test, and the Mann–Whitney U test [54]. Spearman correlation was utilized to assess the statistical dependence between the rankings of two continuous variables, which was expressed as a correlation coefficient ρ.

Univariate analyses were used to identify variables with a good discriminatory value for the binary variables that define resilience. To assess the predictors of resilience, the continuous variable BRS score was converted into a binary variable using the median split and a split within the 75th percentile. The following demographic predictors were used: age ≥ 25 years (co: age < 25 years), having a stable partnership (co: not having a stable partnership), and educational level indicating a tertiary level of education (co: up to secondary education level). The age cut-off was selected according to previous data showing increased psychological distress in the population aged 24 years and under [55]. The following pandemic-specific variables were used: a large reduction in social network (co: no reduction or mild reduction), a period of social distancing ≥15 days (co: a period of social distancing <15 days), isolation or quarantine (co: not being in isolation or quarantine), and a reduction in social support (co: no reduction in social support). The cut-off value of 15 days for the period of social distancing was chosen in accordance with previously published data on the duration of imposed quarantine [56]. Regarding predictors for disease-specific variables, the following items were included: duration since endometriosis diagnosis (in years, continuous variable); age at endometriosis diagnosis (in years, continuous variable); duration since pain onset (in years, continuous variable); diagnostic delay (in years, continuous variable); continuous pain (co: patients with pain peaks); and number of pain localizations (continuous variable, six localizations were assessed). Pain intensity prior to isolation or quarantine as well as current pain levels were considered, in the form of dysmenorrhea, non-cyclic pain, dyspareunia, dyschezia, dysuria, lower back pain, and general pain (mean pain intensity with regard to all previously stated pain localizations).

Variables with *p*-values less than 0.25 in the univariate regression model were subsequently entered into the final model multivariate logistic regression by backward stepwise selection to assess the independence of the variables mentioned above for predicting resilience [57,58]. The odds ratio (OR), variance (Nagelkerke R^2^), *p*-value, and 95% confidence interval (95% CI) were employed to express the data. The effect size (ES) was computed following the method published by Chinn S. [59].

All tests were two-tailed and with a significance level of *p* < 0.05. The analyses were carried out using Version 24.0 of IBM SPSS Statistics for Windows, Armonk, New York, USA.

## 3. Results

### 3.1. Demographic Characteristics of the Study Group

A total of 413 participants met the inclusion criteria and accessed the survey, while 273 (66.10%) completed the BRS questionnaire. To understand the differences between those who answered the questions (group “Respondents”) or those who did not (group “Non-respondents”), the demographic and clinical characteristics of both groups were evaluated as displayed in Table 1. There were no statistically significant differences between the demographic and pandemic-specific variables between the two groups. Respondents showed significantly higher VAS scores regarding current dysmenorrhea, dyschezia, and global pain. Concerning pain-induced disability, participants illustrated significantly superior levels of current disability (recreational and professional activities) as well as higher levels in the discretional activities sub-scores and the current global PDI score than non-respondents. As depicted in Table 1, the median resilience level of our study population was 2.66 and the mean 2.75 (SD = 0.82), which was significantly lower (*p* < 0.001) than in a previously published study of healthy adults from the German Gutenberg Brain Study or a representative survey of the German population [51]. The resilience level in our study was significantly less than in previously published subgroups of participants with no preexisting conditions or even with preexisting conditions, such as cardiac rehabilitation patients [17]. Women with diagnosed fibromyalgia showed no significant difference in resilience score (*p* = 0.07) when compared with our population [17]. To ensure the significance of these results, post hoc analyses employing a two-group Satterthwaite t-test with a 0.05 two-sided significance level revealed a power of 99% for the detection of significantly decreased resilience levels compared with the German normative populations as described by Chmitorz and colleagues [51].

### 3.2. Identification of Predictors of Resilience

Univariate logistic regression analyses were used to examine the predictability of selected independent variables on the odds of having a high level of resilience (BRS score > median of the study group) or very high levels of resilience (BRS score >75th percentile of the study group).

The influence of demographic variables on the resilience scores is detailed in Table 2. High educational levels promoted resilience in both considered subgroups (explained variance 9.1% for BRS > 2.66 and 5.4% for BRS > 3.33, respectively). The age of participants, a stable relationship, or living alone had no significant impact on the modification of resilience. None of the following pandemic-specific variables were found to influence the resilience of the participants in our study group: duration of social network reduction, being in isolation or quarantine or a reduced level of the social network. In contrast, the perceived decrement in social support while experiencing pain resulted in significantly lower levels of resilience. Those who perceived a decline in social embeddedness had 55.8% and 71.9% lower odds of scoring >2.66 and >3.33 on the BRS scale, respectively (see Table 3). The perceived loss of social connection accounted for 5.0 and 8.3% of the variance in BRS scores greater than 2.66 and 3.33, respectively.

Table 4, Table 5 and Table 6 represent how endometriosis-related medical history, pain intensity, and pain-induced disability influenced resilience. Diagnostic delay, duration since pain onset, duration since endometriosis diagnosis, or having continuous pain did not affect resilience in endometriosis patients (Table 4). As shown in Table 5, resilience was not associated with pain intensity prior to the pandemic but was negatively linked with increasing levels of current dyspareunia and global pain experience. High levels of current global pain-induced disability were also found to be negatively related to resilience scores (Table 6), while pain-induced disability prior to the pandemic was not (Table 6).

High scores of anxiety, depression, and mental symptom burden were negatively associated with resilience scores (Table 7). The odds of achieving BRS levels higher than 2.66 and 3.33, respectively, were 84.3 and 92.7% lower for those who scored ≥5 on the GAD-2 scale, and 69.2 and 76.4% lower for those participants who scored ≥5 on the PHQ-2 depression scale (Table 7). Thus, high anxiety proved to be a more significant risk factor for low resilience than high scores on the depression scale. The higher the total mental burden was, as measured by the PHQ-4 scale (continuous variable), the lower the odds of scoring >2.66 and >3.33 on the resilience scale (23.1% and 29.2%, respectively). Adverse mental outcomes accounted for 18.5% of the variance in BRS > 2.66 and 21.1% of the variance regarding BRS >3.33 (PHQ-4 scale continuous variable).

Multivariate logistic regression analysis was performed with the strongest predictors of resilience in univariate analyses (*p* < 0.25) in order to evaluate the independence of the predicting variables stated above. Current global pain-induced disability was included in the multivariate analysis to avoid model overfitting and minimize information redundancy. We decided not to include current pain intensity, as correlation analyses revealed highly significant positive correlations between current global pain-induced disability and current pain intensity, such as current dysmenorrhea (ρ = 0.463; *p* < 0.001), current non-cyclic pain (ρ = 0.473; *p* < 0.001), current dyspareunia (ρ = 0.350; *p* < 0.001), current dysuria (ρ = 0.401; *p* < 0.001), current dyschezia (ρ = 0.474; *p* < 0.001), current lower back pain (ρ = 0.445; *p* < 0.001), and current global pain intensity (ρ = 0.600; *p* < 0.001).

The multivariate logistic regression model for participants scoring BRS > 2.66 included having a stable relationship, educational level, duration of social network reduction, perceived reduction in social support, number of pain localizations, current global pain-induced disability, and PHQ-4 score. A high educational level favored high resilience (OR 2.776; 95% CI 1.496–5.153; *p* = 0.001; ES = 0.454), while high mental health symptom burden was negatively associated with resilience (OR 0.798; 95% CI 0.728–0.876; *p* < 0.001; ES = −0.125). Low perceived social support for pain was the most important independent risk factor (OR 0.541; 95% CI 0.307–0.952; *p* = 0.033; ES = −0.339). The final multivariable logistic regression model (n = 261) explained 24.8% of the variance and had a 69.7% sensitivity for predicting resilience levels greater than 2.66 on the BRS questionnaire in endometriosis patients.

Educational level, duration of social network reduction, perceived decrease in social support, current global pain-induced disability, and PHQ-4 score were identified as potential predictors for women scoring in the highest quartile of the ultra-short BRS scale in this study population (BRS > 3.33). The multivariate logistic regression model included 261 participants. High educational level supported high resilience, but this was not statistically significant (OR 2.223; 95% CI 0.977–5.056; *p* = 0.057; ES = 0.441). A high burden of mental health symptoms was negatively associated with resilience (OR 0.701; 95% CI 0.614–0.801; *p* < 0.001; ES = −0.196). Low perceived social support was the most important risk factor for low resilience (OR 0.397; 95% CI 0.189–0.832; *p* = 0.014; ES = −0.510). The final multivariable logistic regression model explained 28.1% of the variance and demonstrated a sensitivity of 75.9% for predicting resilience levels greater than 3.33 on the BRS questionnaire in patients with endometriosis.

## 4. Discussion

To our knowledge, this is the first study to determine the level of resilience and assess the complex associations between demographic, disease-specific, COVID-19 pandemic-specific, and psychological factors of resilience in endometriosis patients. For this purpose, the BRS questionnaire was used to target those at the highest risk for preventive efforts. The BRS survey is an ultra-brief and comprehensible report screening instrument designed to improve the detection of people with low resilience by physicians, even in settings with limited time resources.

The results of the study show that the mean resilience score of patients with endometriosis was, in general, lower than in a group of the German population [51] or in the original study published by Smith et al., except for a subgroup of patients with fibromyalgia, another chronic pain disease [17]. A similar evaluation of affect regulation in women with fibromyalgia described a lower level of positive affect as well as a lack of psychological immunity in this collective compared with women with osteoarthritis [60]. A positive affect was recognized as a source of resilience in women suffering from chronic pain, while a negative affect was linked to adverse mental health outcomes [19,22]. The depletion of coping resources and positive affect resulting from the stress-triggering chronic pain condition, as reported for women with fibromyalgia [60], without adequate time or opportunity for proper the recovery of the individuals’ buffering systems towards stress, might explain why endometriosis patients are also susceptible to low resilience. Therefore, subgroups of chronic pain patients, such as those with fibromyalgia [60] or women with endometriosis, may have a unique vulnerability of the positive affect system to life stressors. Moreover, our findings are potentially associated with endometriosis patients’ beliefs about how well they can bounce back, rather than a lower ability to bounce back.

In this study, we had the excellent opportunity to assess the impact of both internal (e.g., pain or pain-induced disability) and external (e.g., COVID-19 pandemic and social support) stressors on resilience in patients with endometriosis. We were unable to determine whether the low resilience levels in our study population were caused by the long-term effect of the pre-existing chronic pain disease, known as emergent resilience [47], or caused by concurrent changes in everyday life caused by the pandemic. With this investigation, we found a variety of current changes resulting from the pandemic’s impact, including current pain intensity, current pain-induced disability, and current changes in mental health, which may affect so-called minimal-impact resilience [47]. It is vital to evaluate both emergent and minimal-impact resilience to predict a person’s ability to bounce back from stress, since these types of resilience may influence vulnerability to future stressors [16].

Demographic factors were recognized as predictors of resilience. Age is a consistent predictor for resilience, as younger children appear to exert lower resilience, while adults have often internalized mechanisms to cope with stressful events [47]. However, in our study, age could not be identified as a predictor of resilience. This result may be attributed to the age group studied, as only women over the age of 18 were included. Additionally, female gender is a well-known risk factor for low resilience [47], which may help to explain the low resilience level in our study group. Univariate analyses showed that higher educational levels had a significant positive effect on resilience in women with endometriosis. When other factors were taken into account, a high educational level was found to be a moderator of resilience. This could be related to the fact that it was an independent protective factor only in women with BRS scores higher than the median but not in women with resilience scores in the highest quartile group. The results tied well with the previous findings that a high education level, but not necessarily a higher socioeconomic status, was positively associated with greater resilience [47]. These findings are significant because, compared with other factors, the educational level has general stability over the course of a lifetime.

Pain is a powerful stressor, and chronic pain conditions and resilience seem to mutually influence one another [16,61]. In addition, Zautra et al. reported that pain level could influence the level of positive affect [22]. It is interesting that relatively stable endometriosis-specific factors, such as age at diagnosis or time to pain onset as well as diagnostic delay, had no impact on the resilience of the respondents. In contrast, the age of evolution of endometriosis was positively associated with resilience, as measured by the CDRISC-25 scale [45]. Past stressors, such as past perceived pain or disability, did not predict resilience in the group evaluated. Previous observations have linked a self-perception of a poor-state of health to an insufficient level of resilience to upcoming events [16]. In contrast, in univariate analyses, the level of resilience in the previous two weeks was predicted via current stressors, such as the current level of pain (dyspareunia and global pain experience) and current global pain-induced disability, but not by past stressors, such as pain intensity prior to the pandemic. These results support the findings in the recent literature, which suggest that a higher level of resilience is associated with lower sexual pain [45], and that psychological distress and sexual dysfunction mutually influence each other [62,63]. Bonanno suggested possible changes in resilience over time [64]. These findings imply that resilience in patients with endometriosis is not a stable state of mind or a trait but rather a fluctuant, dynamic process that appears to be influenced by the stressor itself. Nevertheless, when psychological outcomes and perceived social support were taken into account in multivariate analyses, current adverse physical consequences, such as current global pain-induced disability, lost their significance to predict resilience.

During the first wave of the COVID-19 pandemic in Germany, pandemic-specific factors such as duration or level of social distancing and a reduction in social network were not associated with the resilience level in women with endometriosis. This is astounding, as previous findings displayed that cultivating relationships and being a part of a community increased resilience [16]. The missing negative effect of the duration of social distancing in the study group may partly be due to the relatively short length of the period of social network reduction. Moreover, it is likely that the consequences, rather than the duration, of public-health measures to contain viral spread in terms of perceived social connectedness have the potential to alter resilience in women with endometriosis.

Social support is a broad concept that refers to the quality and function of social relationships [65]. Social capital may manifest as social interactions, emotional support, material support, or cognitive support, and can be provided by a variety of systems [65]. Perceived increased social support regarding pain experience, provided by the partner, family, and/or friends, proved to be the strongest independent influencing factor of resilience. These findings are highly significant, as social support may be negatively affected not only as a result of contingency measures due to the pandemic [7], but also by various life adversities within the respective women’s social networks, such as interpersonal conflicts [60,66]. A previous study showed that the level of perceived social stress was inversely associated with the rate of positive affect in women with fibromyalgia, another chronic pain condition [60]. Perceived social support may be related to the extent of one’s social network; thus, women with endometriosis may be at a disadvantage and more susceptible to social stressors because they face life adversities with fewer social resources than healthy controls [44]. In addition, perceived social support reflects the individual’s perception and is only weakly correlated with the actual quantity or quality of the support received [67]. Since social embeddedness is frequently rated and appraised in terms of current needs, which depend on the specific experienced life event or stressor [65,68], a continuous investigation of actual demands and necessities by health-care professionals would be beneficial for women with endometriosis. Social support could influence individual resilience, as well as family and community resilience, and vice versa [65].

Social support is an essential resource for assisting people, especially women, in overcoming adversity, such as natural disasters [69,70], or, in this case, life adversities due to pain. As a result, it is not surprising that functional outcomes were better in people suffering from chronic pain who had good social support systems than in people who experienced limited social interactions [65,66,71]. After a 12-month follow-up of people suffering from pain after traffic injuries, dissatisfaction with social life was recognized as a predictor of a non-resilient mental health trajectory [30]. Similar studies reported that social support buffered the negative effects of life adversities [65,70] and stressors on health and revealed a reduction in mortality of up to 50% in those with satisfying social relationships [72,73]. Social isolation has been linked to (epi-)genetic changes in humans, exposing affected individuals to long-term stress-related alterations in mental health and, thus, resilience [74,75]. According to the results, continuous, effective, and satisfying social interactions appeared to be a source of resilience and well-being in patients with endometriosis. Furthermore, our findings suggest the necessity of a social network with high levels of social support in aiding women with endometriosis to maintain good mental and physical health in the face of stressful life events.

Aside from the importance of social connection in preserving mental well-being, the second most vital finding was the significant association of mental stress and resilience in women with endometriosis. The current psychological burden had a negative and independent impact on resilience; specifically, high anxiety levels had a greater negative impact than severe depression levels. In univariate analyses, mental health (as measured by PHQ-4) accounted for 18.5 to 21.1% of the variance in resilience in both groups, BRS > 2.66 and BRS > 3.33, respectively. Multivariate analyses confirmed that poor mental health and resilience were inversely correlated in patients with endometriosis when other potential influential factors were adjusted for. Scoring >9 on the PHQ-4 scale had 76.9% and 88.4% lower odds of scoring >2.66 and >3.33 on the BRS scale, respectively. During the COVID-19 pandemic, or other natural or man-made disasters, the inverse relationship between mental health and resilience was extensively studied and validated in various populations [24,26,27,28]. These studies described an inverse correlation between positive and negative affect in women suffering from chronic pain conditions such as rheumatoid arthritis, osteoarthritis, or fibromyalgia [22,76]. Additionally, Zautra et al. found that a negative affect was inversely correlated with positive affect and was simultaneously moderated by pain intensity [22].

### Limitations

First, an online survey was used to recruit participants, possibly resulting in a self-selection effect, as those with the highest resilience level may have responded to the survey. However, a recent systematic review confirmed that samples recruited by Facebook were as representative as samples recruited via traditional methods [77]. Nevertheless, social desirability bias was greatly limited because the patients responded directly to the questionnaire.

Second, a significant drop was observed in the sample size of participants, with 413 accessing the survey but only 273 responding to the resilience questionnaire. The high drop-out rate may be related to a bias, as the patients who completed the questionnaire may be those who were experiencing a greater degree of pain or psychological burden at the time of the survey, or who had a greater interest in the topic. We noticed several significant differences in evaluated clinical characteristics, such as current dysmenorrhea, dyschezia, or global pain-induced disability, between non-respondents and those who responded to the questions with respect to the assessed variables. Nevertheless, none of the factors listed above were independent predictors of resilience level. Subsequently, the observed differences between respondents and non-respondents did not affect the study’s outcomes. We did not assess the impact of stage of endometriosis on resilience, but as the stage of endometriosis does not correlate with symptoms, treatment response, or prognosis, we did not expect any impact of the stage of disease on the level of resilience [1]. Third, as the current results are based on cross-sectional research, we were unable to investigate the potential relationship between the emergent stressor and the change in resilience levels in response to the development of the COVID-19 pandemic. Even so, the study’s design had no impact on the results because pandemic-specific factors did not influence resilience levels.

## 5. Conclusions

We showed that adverse mental health outcomes, specifically, self-reported depression or anxiety, and resilience mutually influenced one another. Resilience was influenced by current life events, current mental health, or low perceived social support, and was shown to be more of a process than a constant, non-changeable trait. Since resilience can restore a person’s equilibrium after adverse chronic or acute life events, we recommend the evaluation of resilience in patients with endometriosis on a regular basis (at diagnosis and throughout treatment and follow-up). Women with endometriosis who additionally experience low resilience might benefit from resilience-building measures, resilience-training programs, or interventions to strengthen existing resilient characteristics. The social network of patients with endometriosis should be sensitized, preferably directly after diagnosis, owing to the fact that social support is critical to the well-being of women suffering from chronic pain. Furthermore, public health initiatives should target people who lack or have limited social support to maintain well-being during adversity. Resilience advancement and a reduction in adverse mental outcomes among women with chronic pain conditions should be considered a public health responsibility.

## Figures and Tables

**Table 1:** Differences between participants who did not complete (group “Non-respondents”) versus those who completed the BRS questionnaire (group “Respondents”).

Variables	Values	Non-Respondents	Respondents	*p*-Value
Demographic variables				
Age	<25 y in % (n/N)	13.3% (16/120)	16.5% (45/273)	0.427 ^1^
	≥25 y in %(n/N)	86.7% (104/120)	83.5% (228/273)	
Having a stable relationship	No in % (n/N)	74.5% (79/106)	76.9% (210/273)	0.623 ^1^
	Yes in % (n/N)	25.5% (27/106)	23.1% (63/273)	
Living alone	No in % (n/N)	75.2% (91/121)	79.4% (216/272)	0.352 ^1^
	Yes in % (n/N)	24.8% (30/120)	20.6% (56/272)	
Educational level	Up to secondary level in % (n/N)	50.0% (1/2)	29.2% (78/267)	0.502 ^3^
	Tertiary level in % (n/N)	50.0% (1/2)	70.8% (189/267)	
Pandemic-specific variables				
Duration of i/q	<15 d in % (n/N)	16.2% (17/105)	10.3% (28/273)	0.111 ^1^
	≥15 d in % (n/N)	83.8% (88/105)	89.7% (245/273)	
Being in i/q	No in % (n/N)	5.8% (7/120)	2.6% (7/273)	0.107 ^1^
	Yes in % (n/N)	94.2% (113/120)	97.4% (266/273)	
Reduction in social network	No to moderate reduction in % (n/N)	32.1% (34/106)	27.1% (74/273)	0.336 ^1^
	Large reduction in % (n/N)	67.9% (72/106)	72.9% (199/273)	
Perceived reduction in social support regarding pain experience during social isolation (by partner/family/friends)	No in % (n/N)	75.0% (3/4)	61.4% (164/267)	0.999 ^3^
	Yes in % (n/N)	25.0% (1/4)	38.6% (103/267)	
Endometriosis-specific variables				
Time since diagnosis (y)	M (SD); N	4.02 (4.82); 99	4.38 (4.77); 272	0.289 ^2^
	Mdn (IQR)	2.00 (1.00–5.00)	3.00 (1.00–5.00)	
Age at diagnosis (y)	M (SD); N	28.15 (6.76); 99	27.64 (6.23); 272	0.383 ^2^
	Mdn (IQR)	28.00 (23.00–33.00)	27.00 (23.00–32.25)	
Time since pain onset (y)	M (SD); N	13.34 (7.58); 101	13.99 (7.89); 273	0.520 ^2^
	Mdn (IQR)	12.00 (7.00–18.00)	13.00 (8.00–20.00)	
Diagnostic delay (y)	M (SD); N	9.35 (6.97); 99	9.64 (6.87); 272	0.822 ^2^
	Mdn (IQR)	9.00 (4.00–14.00)	9.00 (4.75–14.00)	
Pain characteristics	Pain peaks in % (n/N)	63.0% (63/100)	65.6% (179/273)	0.645 ^1^
	Continuous pain in % (n/N)	37.0% (36/100)	34.4% (95/273)	
Number of pain localizations	M (SD); N	5.20 (0.88); 44	5.04 (1.199; 271	0.781 ^2^
	Mdn (IQR)	5.00 (5.00–6.00)	5.00 (5.00–6.00)	
Pain intensity				
Dysmenorrhoea prior to i/q	M (SD); N	63.00 (34.30); 42	65.40 (30.82); 242	0.882 ^2^
	Mdn (IQR)	72.50 (34.00–99.00)	73.50 (47.00–89.00)	
Non-cyclical pain prior to i/q	M (SD); N	45.93 (29.64); 44	51.97 (26.77); 259	0.153 ^2^
	Median (IQR)	41.00 (24.00–67.50)	51.00 (32.00–73.00)	
Dyspareunia prior to i/q	M (SD); N	41.77 (34.42); 39	44.87 (32.33); 245	0.590 ^2^
	Mdn (IQR)	30.00 (10.00–70.00)	46.00 (14.00–69.00)	
Dysuria prior i/q	M (SD); N	24.46 (30.81); 39	29.43 (29.06); 240	0.175 ^2^
	Mdn (IQR)	8.00 (1.00–47.00)	20.50 (4.00–48.50)	
Dyschezia prior to i/q	M (SD); N	35.49 (31.90); 41	41.34 (30.90); 252	0.217 ^2^
	Mdn (IQR)	24.00 (9.00–58.00)	37.00 (13.00–67.50)	
Lower back pain prior to i/q	M (SD); N	56.26 (31.91); 43	57.52 (32.51); 264	0.721 ^2^
	Mdn (IQR)	54.00 (33.00–90.00)	61.00 (33.00–87.50)	
Global pain prior to i/q	M (SD); N	43.22 (20.24); 35	47.84 (19.04); 202	0.169 ^2^
	Mdn (IQR)	45.50 (24.83–59.83)	47.83 (33.67–62.00)	
Current dysmenorrhoea	M (SD); N	39.09 (36.84); 11	60.74 (33.48); 246	**0.043 ^2^**
	Mdn (IQR)	39.00 (0.00–80.00)	70.50 (32.00–88.00)	
Current non-cyclical pain	M (SD); N	46.92 (32.10); 12	52.73 (30.26); 262	0.523 ^2^
	Mdn (IQR)	45.00 (22.00–71.50)	56.00 (27.00–78.00)	
Current dyspareunia	M (SD); N	39.55 (38.49); 11	44.24 (35.37); 246	0.700 ^2^
	Mdn (IQR)	22.00 (3.00–79.0)	45.00 (8.00–73.00)	
Current dysuria	M (SD); N	9.09 (13.27); 11	30.08 (31.25); 245	0.065 ^2^
	Mdn (IQR)	5.00 (1.00–10.00)	16.00 (2.00–52.00)	
Current dyschezia	M (SD); N	17.09 (22.10); 11	41.17 (32.46); 250	**0.013 ^2^**
	Mdn (IQR)	4.00 (2.00–36.00)	39.50 (10.00–68.00)	
Current lower back pain	M (SD); N	52.27 (35.82); 11	58.65 (34.04); 260	0.588 ^2^
	Mdn (IQR)	43.00 (24.00–82.00)	64.00 (27.50–88.50)	
Current global pain	M (SD); N	33.20 (20.54); 11	47.22 (21.20); 214	**0.043 ^2^**
	Mdn (IQR)	32.67 (14.67–54.00)	46.58 (32.00–62.67)	
Pain-induced disability				
Family prior to i/q	M (SD); N	5.60 (2.31); 42	5.14 (2.49); 272	0.315 ^2^
	Mdn (IQR)	5.50 (4.00–8.00)	5.00 (3.00–7.00)	
Recreational prior to i/q	M (SD); N	5.45 (2.60); 42	5.61 (2.56); 272	0.714 ^2^
	Mdn (IQR)	6.00 (4.00–7.50)	6.00 (4.00–8.00)	
Social activities prior to i/q	M (SD); N	5.52 (2.85); 42	5.46 (2.73); 272	0.901 ^2^
	Mdn (IQR)	6.00 (3.00–8.00)	6.00 (3.00–8.00)	
Occupational prior to i/q	M (SD); N	6.38 (2.56); 42	5.98 (2.82); 272	0.466 ^2^
	Mdn (IQR)	7.00 (4.00–8.00)	6.00 (4.00–8.00)	
Sexuality prior to i/q	M (SD); N	5.83 (3.18); 42	6.05 (3.27); 267	0.637 ^2^
	Mdn (IQR)	6.00 (3.00–8.00)	7.00 (3.00–9.00)	
Self-care prior to i/q	M (SD); N	2.83 (3.03); 42	2.72 (2.79); 272	0.991 ^2^
	Mdn (IQR)	1.50 (0.00–5.00)	2.00 (0.00–5.00)	
Life support prior to i/q	M (SD); N	2.62 (2.82); 42	2.68 (2.62); 272	0.654 ^2^
	Mdn (IQR)	2.00 (0.00–5.00)	2.00 (0.00–5.00)	
Discretional activities prior to i/q	M (SD); N	28.79 (10.58); 42	28.18 (11.21); 267	0.901 ^2^
	Mdn (IQR)	30.00 (23.00–37.00)	30.00 (21.00–37.00)	
Basic activities prior to i/q	M (SD)	5.45 (5.34); 42	5.41 (4.88); 267	0.744 ^2^
	Mdn (IQR)	5.00 (0.00–9.00)	4.00 (1.00–9.00)	
Global PDI prior to i/q	M (SD); N	34.24 (14.36); 42	33.59 (14.37); 267	0.790 ^2^
	Mdn (IQR)	33.00 (26.00–45.00)	34.00 (23.00–43.00)	
Current family activities	M (SD); N	4.00 (1.83); 0	5.35 (2.72); 271	0.088 ^2^
	Mdn (IQR)	4.50 (2.00–6.00)	5.00 (3.00–8.00)	
Current recreational activities	M (SD); N	3.30 (2.11); 10	5.39 (2.91); 271	**0.019 ^2^**
	Mdn (IQR)	4.00 (1.00–5.00)	6.00 (3.00–8.00)	
Current social activities	M (SD); N	2.80 (2.62); 10	4.57 (3.44); 270	0.085 ^2^
	Mdn (IQR)	3.50 (0.00–5.00)	5.00 (1.00–8.00)	
Current occupational activities	M (SD); N	3.10 (1.97); 10	5.37 (3.27); 270	**0.032 ^2^**
	Mdn (IQR)	4.00 (2.00–5.00)	5.00 (3.00–8.00)	
Current sexuality	M (SD); N	3.90 (3.18); 10	5.58 (3.50); 267	0.126 ^2^
	Mdn (IQR)	3.50 (2.00–5.00)	6.00 (3.00–9.00)	
Current self-care	M (SD); N	2.10 (2.13); 10	2.86 (2.87); 271	0.556 ^2^
	Mdn (IQR)	1.50 (0.00–3.00)	2.00 (0.00–5.00)	
Current life support	M (SD); N	1.90 (2.51); 10	2.69 (2.79); 271	0.326 ^2^
	Mdn (IQR)	0.50 (0.00–3.00)	2.00 (0.00–5.00)	
Current discretional activities	M (SD); N	17.10 (9.64); 10	26.38 (12.52); 267	**0.022 ^2^**
	Mdn (IQR)	20.00 (9.00–26.00)	27.00 (16.00–36.00)	
Current basic activities	M (SD); N	4.00 (4.50); 10	5.62 (5.14); 267	0.305 ^2^
	Mdn (IQR)	2.00 (0.00–6.00)	5.00 (1.00–9.00)	
Current global PDI	M (SD); N	21.10 (12.91); 10	32.00 (15.81); 267	**0.037 ^2^**
	Mdn (IQR)	23.50 (11.00–32.00)	32.00 (19.00–43.00)	
Mental outcomes				
PHQ-2	M (SD); N	n.a.	2.83 (1.69); 272	n.a.
	Mdn (IQR)	n.a.	2.00 (2.00–4.00)	
GAD-2	M (SD); N	n.a.	2.90 (1.83); 272	n.a.
	Mdn (IQR)	n.a.	2.00 (2.00–4.00)	
PHQ-4	M (SD); N	n.a.	5.73 (3.21); 272	n.a.
	Mdn (IQR)	n.a.	5.00 (3.00–8.00)	
Resilience (BRS)	M (SD); N	n.a.	2.75 (0.82); 273	n.a.
	Mdn (IQR)	n.a.	2.66 (2.16–3.33)	

BRS = Brief Resilience Score; GAD-2 = Generalized Anxiety Disorder Scale; PHQ-2 = Patient Health Questionnaire for Depression; PHQ-4 = Patient Health Questionnaire for Depression and Anxiety; i/q = isolation or quarantine; d = days; N = Number of women for which data were available; n = sample size; M= mean; SD = standard deviation, Mdn = median; IQR: Interquartile Range; n.a. = not available/not applicable; y = years. Values in bold indicate statistical significance, as the level of statistical significance was set to *p* < 0.05 (^1^ = χ^2^-test; ^2^ = Mann–Whitney U test; ^3^ = Fisher exact test).

**Table 2:** Influence of demographic factors on resilience (univariate logistic regression analysis).

BRS > 2.66			BRS > 3.33		
*p*-Value	OR (95% CI)	ES	*p*-Value	OR (95% CI)	ES
Age ≥ 25 years (co: <25 years)					
0.535	0.817 (0.431–1.548)	−0.112	0.623	0.833 (0.402–1.726)	−0.101
Having a partner (co: not having a partner)					
0.117	1.573 (0.892–2.771)	0.237	0.500	1.247 (0.656–2.372)	0.122
Living alone (co: not living alone)					
0.392	1.293 (0.718–2.330)	0.142	0.443	0.752 (0.363–1.558)	−0.157
Tertiary educational level (co: up to secondary educational level)					
**<0.001**	**3.393** **(1.906–6.040)**	**0.675**	**0.004**	**3.067** **(1.430–6.577)**	**0.619**

BRS = Brief Resilience Score; BRS > 2.66 = median split; BRS > 3.33 = split > 75th percentile; OR = odds ratio; CI = confidence interval; ES = effect size; co = controls. Values in bold indicate statistical significance, as the level of statistical significance was set to *p* < 0.05.

**Table 3:** Influence of pandemic-specific factors on resilience (univariate logistic regression analysis).

BRS > 2.66			BRS > 3.33		
*p*-Value	OR (95% CI)	ES	*p*-Value	OR (95% CI)	ES
Duration of reduction in social network ≥15 days (co: <15 days)					
0.104	1.994 (0.868–4.580)	0.381	0.219	1.989 (0.664–5.960)	0.379
Being in isolation or quarantine (co: not being in isolation or quarantine)					
0.829	1.182 (0.260–5.384)	0.092	0.765	0.776 (0.147–4.097)	−0.140
Large reduction in social network (co: not at all to moderate reduction in social network)					
0.462	1.223 (0.715–2.094)	0.111	0.605	1.184 (0.624–2.247)	0.093
Perceived reduction in social support during pain experience (co: no reduction in social support)					
**0.002**	**0.442** **(0.267–0.733)**	**−0.451**	**<0.001**	**0.281** **(0.142–0.556)**	**−0.701**

BRS = Brief Resilience Score; BRS > 2.66 = median split; BRS > 3.33 = split > 75th percentile; OR = odds ratio; CI = confidence interval; ES = effect size; co = controls. Values in bold indicate statistical significance, as the level of statistical significance was set to *p* < 0.05.

**Table 4:** Influence of endometriosis-specific history on self-reported mental health outcomes (univariate logistic regression analysis).

BRS > 2.66			BRS > 3.33		
*p*-Value	OR (95% CI)	ES	*p*-Value	OR (95% CI)	ES
Duration since diagnosis of endometriosis (years)					
0.440	1.020 (0.970–1.072)	0.011	0.680	0.987 (0.929–1.049)	−0.007
Age at diagnosis of endometriosis (in years)					
0.942	0.999 (0.961–1.038)	−0.001	0.353	1.022 (0.977–1.069)	0.012
Duration since pain onset (in years)					
0.638	0.993 (0.963–1.023)	−0.004	0.835	0.996 (0.961–1.032)	−0.002
Diagnostic delay (continuous variable in years)					
0.257	0.980 (0.946–1.015)	−0.011	0.990	1.000 (0.960–1.042)	0.000
Pain characteristics: continuous pain (co: pain peaks)					
0.299	0.766 (0.463–1.267)	−0.147	0.477	0.805 (0.442–1.464)	−0.119
Number of pain locations					
0.059	0.821 (0.669–1.007)	−0.109	0.347	0.897 (0.716–1.125)	−0.060

BRS = Brief Resilience Score; BRS > 2.66 = median split; BRS > 3.33 = split > 75th percentile; OR = odds ratio; CI = confidence interval; ES = effect size; co = controls.

**Table 5:** Influence of pain intensity on resilience (univariate logistic regression analysis).

	BRS > 2.66			BRS > 3.33		
	*p*-Value	OR (95% CI)	ES	*p*-Value	OR (95% CI)	ES
Dysmenorrhea prior to i/q						
**VAS_P_**	0.407	0.997 (0.988–1.005)	−0.002	0.357	0.996 (0.986–1.005)	−0.002
Non-cyclic pain prior to i/q						
**VAS_P_**	0.604	1.002 (0.993–1.012)	0.001	0.426	0.996 (0.985–1.006)	−0.002
Dyspareunia prior to i/q						
**VAS_P_**	0.558	0.998 (0.990–1.005)	−0.001	0.716	0.998 (0.989–1.007)	−0.001
Dysuria prior to i/q						
**VAS_P_**	0.708	1.002 (0.993–1.010)	0.001	0.569	0.997 (0.987–1.007)	−0.002
Dyschezia prior to i/q						
**VAS_P_**	0.560	1.002 (0.994–1.010)	0.001	0.795	0.999 (0.989–1.008)	−0.001
Lower back pain prior to i/q						
**VAS_P_**	0.386	0.997 (0.989–1.004)	−0.002	0.384	0.996 (0.987–1.005)	−0.002
Global pain experience prior to i/q						
**VAS_P_**	0.989	1.000 (0.985–1.015)	0.000	0.685	0.997 (0.980–1.013)	−0.002
Current dysmenorrhea						
**VAS_C_**	0.088	0.993 (0.986–1.001)	−0.004	0.154	0.994 (0.985–1.002)	−0.003
Current non-cyclic pain						
**VAS_C_**	0.127	0.994 (0.986–1.002)	−0.003	0.217	0.994 (0.985–1.004)	−0.003
Current dyspareunia						
**VAS_C_**	0.088	0.994 (0.987–1.001)	**−0.003**	**0.027**	**0.990** **(0.982–0.999)**	**−0.005**
Current dysuria						
**VAS_C_**	0.603	1.002 (0.994–1.010)	0.001	0.522	0.997 (0.987–1.006)	−0.002
Current dyschezia						
**VAS_C_**	0.332	0.996 (0.989–1.004)	−0.002	0.072	0.991 (0.982–1.001)	−0.005
Current lower back pain						
**VAS_C_**	0.182	0.995 (0.988–1.002)	−0.002	0.171	0.994 (0.986–1.003)	−0.003
Current global pain experience						
**VAS_C_**	**0.034**	**0.986** **(0.973–0.999)**	**−0.007**	**0.014**	**0.981** **(0.966–0.996)**	**−0.010**

BRS = Brief Resilience Score; BRS > 2.66 = median split; BRS > 3.33 = split > 75th percentile; OR = odds ratio; CI = confidence interval; ES = effect size; i/q = isolation or quarantine; VAS_P_ = previous pain level, continuous variable; VAS_C_ = current pain level, continuous variable; co= controls. Values in bold indicate statistical significance, as the level of statistical significance was set to *p* < 0.05.

**Table 6:** Impact of pain-induced disability on resilience (univariate logistic regression analysis).

	BRS > 2.66			BRS > 3.33		
	*p*-Value	OR (95% CI)	ES	*p*-Value	OR (95% CI)	ES
Pain-induced disability prior to i/q						
Family	0.198	0.939 (0.853–1.034)	−0.034	0.962	0.997 (0.892–1.115)	−0.002
Recreational	0.882	0.993 (0.905–1.090)	−0.004	0.114	1.088 (0.972–1.218)	0.046
Social activities	0.163	0.939 (0.860–1.026)	−0.035	0.642	1.025 (0.924–1.136)	0.014
Occupational	0.209	0.947 (0.870–1.031)	−0.030	0.376	0.957 (0.867–1.055)	−0.024
Sexuality	0.078	0.936 (0.869–1.008)	−0.036	0.822	0.990 (0.909–1.078)	−0.005
Self-care	0.227	0.948 (0.870–1.034)	−0.029	0.330	0.950 (0.856–1.054)	−0.028
Life support	0.625	0.978 (0.892–1.071)	−0.012	0.894	0.993 (0.892–1.105)	−0.004
Discretional activities	0.092	0.982 (0.960–1.003)	−0.010	0.845	1.002 (0.978–1.028)	0.001
Basic activities	0.263	0.972 (0.925–1.022)	−0.016	0.524	0.981 (0.925–1.040)	−0.010
Global PDI; PDI_P_	0.090	0.985 (0.969–1.002)	−0.008	0.949	0.999 (0.980–1.019)	−0.001
Current pain-induced disability						
Family	**0.013**	**0.892** **(0.815–0.976)**	−0.063	0.440	0.960 (0.866–1.064)	−0.022
Recreational	0.178	0.945 (0.870–1.026)	−0.031	0.467	0.956 (0.877–1.062)	−0.025
Social activities	0.087	0.941 (0.877–1.009)	−0.034	0.164	0.943 (0.868–1.024)	−0.032
Occupational	**0.032**	**0.922** **(0.856–0.993)**	**−0.045**	**0.015**	**0.897** **(0.821–0.979)**	**−0.060**
Sexuality	**0.016**	**0.918** **(0.856–0.948)**	−0.047	0.167	0.945 (0.872–1.024)	−0.031
Self-care	0.559	0.975 (0.897–1.061)	−0.014	0.093	0.914 (0.822–1.015)	−0.049
Life support	0.467	0.969 (0.889–1.056)	−0.017	0.326	0.949 (0.854–1.054)	−0.029
Discretional activities	**0.007**	**0.973** **(0.954–0.993)**	−0.015	0.051	0.978 (0.956–1.000)	−0.012
Basic activities	0.470	0.983 (0.938–1.030)	−0.009	0.144	0.958 (0.903–1.015)	−0.023
Global PDI; PDI_C_	**0.018**	**0.981** **(0.966–0.997)**	**−0.011**	**0.043**	**0.981** **(0.963–0.999)**	**−0.011**

BRS = Brief Resilience Score; BRS > 2.66 = median split; BRS > 3.33 = split > 75th percentile; OR = odds ratio; CI = confidence interval; ES = effect size; i/q = isolation or quarantine; PDI_P_ = disability previous to i/q, continuous variable; PDI_C_ = current disability, continuous variable. Values in bold indicate statistical significance, as the level of statistical significance was set to *p* < 0.05.

**Table 7:** Effect of mental health on resilience (univariate logistic regression analysis).

	BRS > 2.66			BRS > 3.33		
	*p*-Value	OR (95% CI)	ES	*p*-Value	OR (95% CI)	ES
PHQ-2 cv	<0.001	0.686 (0.586–0.803)	−0.208	<0.001	0.634 (0.516–0.779)	−0.252
PHQ-2 ≥ 3 (co: PHQ-2 < 3)	<0.001	0.361 (0.220–0.592)	−0.563	<0.001	0.321 (0.169–0.579)	−0.628
PHQ-2 ≥ 5 (co: PHQ-2 < 5)	<0.001	0.302 (0.158–0.575)	−0.661	0.003	0.236 (0.090–0.619)	−0.798
GAD-2 cv	<0.001	0.619 (0.529–0.724)	−0.265	<0.001	0.517 (0.412–0.648)	−0.364
GAD-2 ≥ 3 (co:GAD-2 < 3)	<0.001	0.240 (0.145–0.399)	−0.788	<0.001	0.110 (0.052–0.234)	−1.219
GAD-2 ≥ 5 (co:GAD-2 < 5)	<0.001	0.157 (0.077–0.317)	−1.023	<0.001	0.073 (0.017–0.306)	−1.446
PHQ-4 cv	<0.001	0.769 (0.704–0.839)	−0.145	<0.001	0.708 (0.626–0.801)	−0.191
PHQ-4 ≥ 6 (co: PHQ-4 < 6)	<0.001	0.221 (0.132–0.371)	−0.834	<0.001	0.157 (0.076–0.325)	−1.023
PHQ-4 ≥ 9 (co: PHQ-4 < 9)	<0.001	0.231 (0.120–0.443)	−0.809	<0.001	0.116 (0.035–0.383)	−1.190

BRS = Brief Resilience Score; BRS > 2.66 = median split; BRS > 3.33 = split > 75th percentile; GAD-2 = Generalized Anxiety Disorder Scale; PHQ-2 = Patient Health Questionnaire for Depression; PHQ-4 = Patient Health Questionnaire for Depression and Anxiety; OR = odds ratio; CI = confidence interval; ES = effect size; cv = continuous variable; co = controls. The level of statistical significance was set to *p* < 0.05.

## Data Availability

The datasets analyzed during the current study are available from the corresponding author on reasonable request.
